# The gut-lung axis: effects and mechanisms of gut microbiota on pulmonary diseases

**DOI:** 10.3389/fimmu.2025.1693964

**Published:** 2026-01-05

**Authors:** Jiaxin Liu, Weichen Hong, Zhendong Sun, Shuyu Zhang, Chenyu Xue, Na Dong

**Affiliations:** College of Animal Science and Technology, Northeast Agricultural University, Harbin, China

**Keywords:** gut microbiota, gut-lung axis, immune response, inflammatory process, microbiome intervention

## Abstract

The proposal of the gut–lung axis has profoundly reshaped our understanding of the mechanisms underlying respiratory diseases. As a crucial component of this axis, the gut microbiota plays a central role in pulmonary immune regulation through inter-organ communication mediated by metabolic products. However, a systematic integration of mechanisms explaining how gut microbes achieve precise cross-organ immune regulation remains elusive. Existing research predominantly focuses on descriptive observations, such as the association between early-life microbiota dysbiosis and an increased risk of asthma and chronic obstructive pulmonary disease (COPD), as well as the frequent occurrence of acute respiratory distress syndrome (ARDS) and pulmonary fibrosis (PF), often accompanied by gut microbiome disruption. This paper focuses on three key gut microbial metabolites—short-chain fatty acids (SCFAs), tryptophan metabolites, and polyamines (PAs)—to examine their roles in immune regulation, maintenance of barrier function, and modulation of metabolic signaling networks. Based on the latest experimental and clinical evidence, this study systematically elucidates how dysbiosis of the gut microbiota, a key component of the gut-lung axis, crosses physiological barriers to exacerbate pulmonary inflammation. Regarding intervention strategies, probiotics, fecal microbiota transplantation (FMT), and CRISPR-Cas systems have demonstrated significant therapeutic potential in restoring gut microbial balance. Finally, this paper outlines future research directions, emphasizing the need to further explore non-invasive microbial sampling techniques, molecular interaction mechanisms of the gut-lung axis, and personalized microbiome-based diagnostic and therapeutic strategies to provide new insights for the prevention and treatment of respiratory diseases involving gut microbiota.

## 1 Introduction

Throughout the evolutionary journey of human life, the human body and microorganisms have coexisted in a mutual relationship of dependence and influence. Microbial communities colonize various parts of the body, primarily residing in the gastrointestinal tract and respiratory tract (1–3). They play crucial roles in the development of the human immune system, the regulation of metabolic balance, and disease prevention (4). Among these, the gut microbiota represents the most abundant and diverse microbial community within the human body, comprising bacteria, fungi, viruses, and other microorganisms. Its functions manifest in two primary aspects. Firstly, it serves as a vital agent for breaking down dietary fiber. During this process, it releases small-molecule metabolites (5) that regulate digestion and absorption while maintaining immune homeostasis (6). For instance, enzymes such as cellulase and pectinase, secreted by microbes, break down dietary fiber into oligosaccharides. These oligosaccharides undergo further fermentation by microbes in the colon, ultimately yielding small-molecule metabolites, primarily short-chain fatty acids (SCFAs). This process also produces other bioactive compounds such as indoles and polyamines (PAs), which serve as signaling molecules for subsequent digestive regulation and immune maintenance. On the other hand, gut microbes can also exert direct effects on immune regulation within the intestine. They modulate immune responses in distant mucosal sites—such as pulmonary mucosal immunity—through mechanisms including immune cascades and immune migration (7).

The gut and lungs are both key organs directly exposed to the external environment, interconnected through a complex bidirectional communication network that forms the gut-lung axis. This concept challenges the traditional view of the digestive and respiratory systems as independent entities, revealing how the gut microbiota plays a pivotal role in the pathophysiology of lung diseases by regulating immune and metabolic homeostasis (8). The bidirectionality of this axis manifests as follows: early-life antibiotic-induced gut dysbiosis increases the risk of allergic airway diseases (9–12). Conversely, pulmonary diseases such as asthma and chronic obstructive pulmonary disease (COPD) are frequently associated with microbial dysregulation in both the airways and the gastrointestinal tract (13, 14).

Early evidence of gut-lung interactions emerged from clinical observations, such as the significantly elevated incidence of chronic bronchopulmonary diseases in patients with inflammatory bowel disease (IBD) (15–17). A large-scale cohort study revealed that patients with COPD had a 2.72-fold higher risk of Crohn’s disease (CD) compared to healthy controls, often accompanied by specific intestinal manifestations, such as malabsorption in the small intestine (18–20). Similarly, pulmonary involvement—including interstitial lung disease, alveolar lymphocytosis, and reduced pulmonary diffusion capacity—is frequently detected in IBD patients (21), with respective prevalence rates of 44% (22), 48% (23), and 50% (24). Furthermore, clinical studies reveal that patients with idiopathic pulmonary fibrosis (IPF) undergoing pirfenidone therapy experience significantly increased gastrointestinal disease risk when consuming high saturated fatty acid (SFA) diets (25). These findings collectively validate the close pathophysiological connection between the gut and lungs. The recent discovery of the gut commensal protozoan *Tritrichomonas musculis* (T.mu) (26) has provided a novel perspective on how gut microbiota remotely regulate the immune system (27). The discovery of a novel subtype of type II innate lymphoid cells (ml-ILC2s) not only provides new insights into elucidating the pathophysiological mechanisms of asthma but also contributes crucial evidence to the study of mechanisms within the gut-lung axis (28). The gut-lung axis plays a pivotal role in multiple diseases, offering fresh insights into unraveling the mysteries of human health and tackling disease challenges.

However, current research faces significant limitations: most studies remain fragmented interpretations of single pathways and effects, with insufficient extrapolation from animal models to human clinical settings. For instance, commonly used mouse asthma models (e.g., OVA sensitization) can only mimic a single subtype of allergic asthma, failing to replicate the heterogeneity of human asthma and its complex associations with gut microbiota, environmental exposures, and other multifactorial influences (29, 30). This limitation explains why drugs targeting airway inflammation demonstrate clinical efficacy only in a subset of patients. This review aims to comprehensively analyze existing research findings to explore the mechanisms by which the gut microbiota functions as a critical component of the gut-lung axis, thereby providing new therapeutic directions for related diseases. Additionally, we will discuss future research directions and challenges, providing insights for further investigation into the mechanisms by which the gut microbiota influences the gut-lung axis.

## 2 Gut-lung axis

Traditional Chinese medicine theory has long held the view that “the lung and the large intestine are interiorly and exteriorly related” (31). Through multidisciplinary exploration involving molecular biology and chemical bioinformatics, a profound understanding has emerged of the close and inseparable connection between the gut and lungs (32). Research indicates that the gut and lungs share a certain modern biological foundation, with their pathophysiological changes exhibiting a degree of synchrony. Both organs share an embryonic origin in the endoderm of the gastrula (33), establishing a biological foundation for their deep connection. Despite differences in epithelial structure (34), they both harbor rich microbial communities and maintain barrier and microbial balance to defend against pathogens (35). More significantly, they exhibit extensive sharing and interaction in cell types and signaling pathways (36).

At the immunological mechanism level, the mucosal lymphocyte homing theory is central to explaining gut-lung axis. This theory posits that lymphocytes activated in mucosal sites, such as the gut, can express specific homing receptors (e.g., α4β7 integrin) to migrate directionally to distant mucosal sites, such as the lungs, thereby enabling immune coordination. This mechanism is concretely manifested in the production of secretory immunoglobulin A (sIgA): antigen-activated B cells can be precisely recruited to the mucosal lamina propria of another organ via homing pathways, such as CCR10/CCL28 and α4β7/MAdCAM-1. There, they differentiate into plasma cells and produce sIgA, thereby establishing systemic mucosal immune defense (37–39).

Notably, these pathways play distinct roles in immunoglobulin A (IgA) cell homing: The CCR10/CCL28 pathway serves as the primary route for cross-organ homing. IgA-secreting cells highly express CCR10, enabling them to recognize the chemokine CCL28, which is secreted by intestinal and pulmonary epithelial cells. This facilitates directed migration to multiple mucosal tissues, such as the gut and lungs, forming an extensive immune network. whereas the α4β7 integrin/MAdCAM-1 pathway primarily mediates gut-specific homing. Under normal conditions, this pathway predominates in intestinal homing, allowing gut-activated IgA precursor cells to preferentially colonize the intestinal lamina propria. However, under abnormal conditions such as pulmonary inflammation, airway endothelial cells abnormally express MAdCAM-1, attracting IgA cells expressing α4β7 integrin from the gut to migrate to the lungs and participate in local immune responses.

Furthermore, cutting-edge research continues to provide new perspectives on the gut-lung axis mechanism. For instance, in a mouse model of sepsis, gut-derived memory γδ T17 cells migrate to the lungs via the Wnt/β-catenin signaling pathway, releasing IL-17 to exacerbate lung injury. Conversely, dextroketamine (S-KT) reduces cell migration by inhibiting this pathway, offering a novel therapeutic target for sepsis treatment (40). Collectively, these findings demonstrate that intestinal immune cells can remotely regulate pulmonary immune homeostasis through the gut-lung axis (41) and even influence the growth and function of bone marrow cells (42, 43).

From their shared embryonic origin to their common mucosal immune systems, and from fundamental homing pathways to cutting-edge cell migration mechanisms, modern research has revealed profound intrinsic connections between the gut and lungs across multiple dimensions. Therefore, in-depth investigation of gut microbiota holds significant importance for elucidating the mechanisms of the gut-lung axis (32) and guiding clinical diagnosis and treatment (14).

## 3 Mechanisms of gut microbiota influence on the gut-lung axis

The gut is one of the largest immune organs in the human body (44). The gut microbiota exhibits remarkable diversity and richness. In healthy individuals, the gut microbiota forms a complex and dynamically balanced ecosystem primarily dominated by four major bacterial phyla: Firmicutes, Bacteroidetes, Actinobacteria, and Proteobacteria (45). At the genus level, common symbionts include Bacteroides, Prevotella, Ruminococcus, Bifidobacterium, and Lactobacillus. These microorganisms play crucial roles in maintaining intestinal barrier integrity, regulating the immune system, and producing metabolites. Through these functions, gut microbes remotely regulate pulmonary homeostasis. For instance, in germ-free mouse models, compared to wildling mice with richer microbiomes, germ-free mice exhibit more intense inflammatory responses in allergic airway diseases and poorer outcomes during acute infections (46, 47).

The gut primarily signals to the lungs via two circulatory pathways—the blood and lymphatic systems—enabling communication between the gut and the lungs. First, via the portal vein-systemic circulation pathway, microbial metabolites absorbed by the intestinal epithelium (e.g., SCFAs, tryptophan derivatives) are transported to the liver via the portal vein. After partial hepatic metabolism, the remaining active molecules enter the systemic circulation and ultimately reach the pulmonary vasculature. There, they bind to receptors on the cell surfaces, such as those found in alveolar macrophages, to modulate the immune status. In the mesenteric lymphatic-thoracic duct pathway, certain metabolites, antigens, and regulated immune cells are absorbed by intestinal lymphatics and directly injected into the systemic circulation via the thoracic duct. Bypassing the liver, this pathway delivers these signaling molecules and cells to the lungs at higher bioactive concentrations, enabling more precise and effective participation in mucosal immune responses.

It is crucial to emphasize that gut-lung axis is a bidirectional process. Pulmonary inflammation can trigger systemic inflammatory responses and stress states. The released inflammatory cytokines and stress hormones can affect intestinal blood flow, disrupt the epithelial barrier, and subsequently lead to dysbiosis of the gut microbiota. Consequently, harmful substances such as endotoxins (LPS) enter the circulation, further exacerbating systemic inflammation. These substances then feed back to the lungs via the bloodstream, forming a vicious cycle centered on the gut microbiota that continuously worsens lung injury.

Thus, a series of metabolites derived from the gut microbiota serves as the core mediators enabling this bidirectional communication. This paper will now delve into three key metabolites—SCFAs, tryptophan derivatives, and PAs—detailing their specific molecular mechanisms for regulating immune balance through the gut-lung axis.

### 3.1 SCFAs

Diet is one of the most effective strategies in adjunctive therapy for gut dysbiosis, as it can influence gut microbiome dynamics and host health (48). Numerous epidemiological studies indicate a negative correlation between dietary fiber intake and asthma incidence (49, 50). Patients with severe asthma typically have lower fiber intake, while a high-fiber diet can reduce serum levels of inflammatory markers (IL-6 and CRP) (51). This occurs because SCFAs, produced by gut microbes fermenting dietary fiber, enhance the function of regulatory T cells (Tregs), thereby alleviating allergic airway inflammation (52, 53). These findings have been validated in mouse models. Therefore, in-depth investigation of SCFAs’ role and mechanisms in immune regulation (5, 54) holds significant implications for understanding related diseases.

SCFAs are the end products of gut microbial fermentation of dietary fiber, primarily comprising acetate, propionate, and butyrate. After absorption by intestinal epithelial cells, they enter the liver via the portal vein. Following partial metabolism, they enter systemic circulation and ultimately reach the lungs via the bloodstream, enabling gut-lung communication.

**Figure 1:**
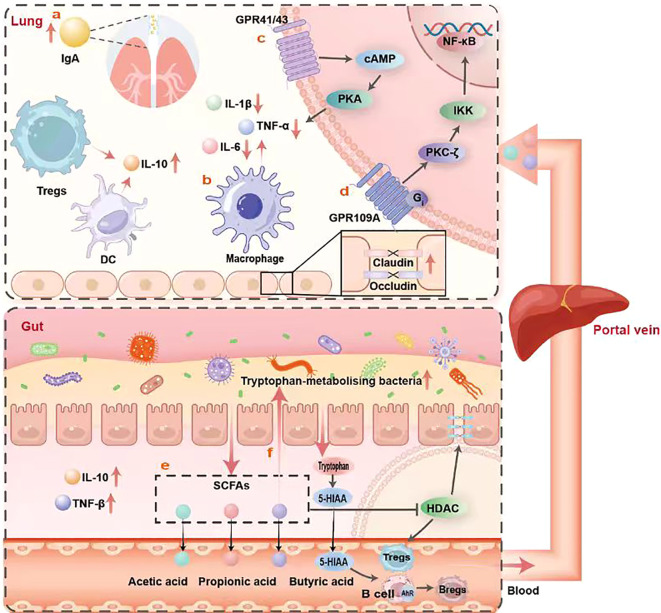
This schematic diagram illustrates the core mechanisms by which SCFAs derived from gut microbiota regulate immune balance along the gut-lung axis through multiple pathways. The diagram is divided into two main sections: the lungs (upper portion) and the gut (lower portion), interconnected via the portal vein and blood circulation. The lung section depicts SCFA-mediated immune regulation mechanisms upon reaching the lungs: primarily by promoting IgA secretion to maintain pulmonary mucosal immune homeostasis **(a)**; modulating alveolar macrophage polarization to enhance epithelial barrier protein function **(b)**; GPR41/43 receptor inhibition of the cAMP-PKA signaling pathway to downregulate proinflammatory factors IL-1β and IL-6 **(c)**; and activating the PKC-ζ-IKK-NF-κB signaling cascade via GPR109A to promote anti-inflammatory mediator expression **(d)**. The intestinal section illustrates how dietary fiber undergoes fermentation by gut microbiota to produce SCFAs (acetic acid, propionic acid, butyric acid). These SCFAs exert effects through the following pathways: promoting Treg differentiation via HDAC inhibition **(e)**; influencing 5-HIAA production by regulating tryptophan metabolism, thereby modulating Breg function through AhR **(f)**; and facilitating the migration of generated Tregs and regulatory immune cells to the lungs via the bloodstream.

SCFAs exert multifaceted regulatory effects on the immune system, primarily manifested in two aspects. Firstly, SCFAs (especially butyrate) serve as the primary energy source for colonic epithelial cells, promoting goblet cell differentiation and mucus secretion, strengthening epithelial tight junctions, and enhancing IgA production (55, 56). The mucus produced by goblet cells and IgA jointly form a protective layer on the intestinal mucosal surface. Mucus physically prevents pathogens from directly contacting intestinal epithelial cells, while IgA specifically binds pathogens, blocking their adhesion and invasion of epithelial cells. Together, they construct a physical and immune barrier (57, 58), effectively preventing pathogen invasion and the translocation of harmful substances (**Figure 1a**). On the other hand, SCFAs play a crucial role in core immunoregulatory functions in the lungs. For instance, acetate promotes the anti-inflammatory polarization of alveolar macrophages, increasing IL-10 secretion while reducing the release of pro-inflammatory factors, such as TNF-α and IL-6 (**Figure 1b**). Mice deficient in SCFAs receptors exhibit more severe inflammatory responses in asthma models (7, 41, 52, 59), confirming their critical role in respiratory tract protection (60).

The direct immunomodulatory effects of SCFAs are mediated through binding to G protein-coupled receptors (GPCRs) such as GPR109A, GPR41, and GPR43, which exhibit differential expression across cell types and tissues (61, 62). Upon ligand binding, GPCRs couple with distinct downstream effectors (Gi/o or Gq), triggering intracellular signaling cascades. For instance, activation of GPR41 and GPR43 inhibits adenylate cyclase (AC) activity (63), thereby reducing intracellular cyclic AMP (cAMP) production and suppressing protein kinase A (PKA) signaling. This mechanism produces multiple anti-inflammatory effects in the lung. In alveolar macrophages, it suppresses the transcription and release of pro-inflammatory cytokines, including TNF-α, IL-1β, and IL-6 (**Figure 1c**). Simultaneously, it influences T cells differentiation by promoting Treg generation and function while inhibiting Th17 cell differentiation, thereby regulating pulmonary immune homeostasis. In pulmonary epithelial cells, it enhances epithelial barrier integrity by regulating the phosphorylation status of tight junction proteins, such as Occludin and Claudin, thereby reducing vascular permeability. This effectively prevents plasma protein and inflammatory cell extravasation, alleviates pulmonary edema, and protects gas exchange function. For instance, following respiratory syncytial virus infection, acetate activates GPR43 to modulate the interferon-β (IFN-β) response in pulmonary epithelial cells (64) and stimulates the proliferation of colonic lumen-associated Tregs, leading to substantial production of the anti-inflammatory cytokine IL-10 (33). Furthermore, GPR109A, upon activation by butyrate, similarly inhibits cAMP production via Gi protein coupling, thereby activating protein kinase C-ζ (PKC-ζ). Activated PKC-ζ phosphorylates IκB kinase (IKK), leading to IκB phosphorylation and degradation, thereby releasing nuclear factor κB (NF-κB). Upon entering the nucleus, NF-κB promotes the differentiation and proliferation of Tregs by initiating the transcription of specific genes (**Figure 1d**). These Tregs suppress effector T cell activity by secreting cytokines such as IL-10. Simultaneously, the activation of transcription factors like NF-κB suppresses macrophage overactivation, thereby reducing their release of proinflammatory cytokines, such as TNF-α and IL-1β.

Furthermore, SCFAs exert immunomodulatory effects through histone deacetylase (HDAC) inhibition pathways (65, 66). After entering cells via passive diffusion or specific transporters such as the high-affinity Na^+^-coupled SLC5A8 transporter or low-affinity H^+^-coupled SLC16A1 transporter, SCFAs effectively inhibit HDAC activity (14). This leads to increased histone acetylation, loosening chromatin structure and facilitating transcription factor binding to DNA. Consequently, gene expression related to immune regulation, cell differentiation, and inflammatory responses is activated. In the gut, SCFAs promote the differentiation and function of Tregs while suppressing the production of proinflammatory cytokines. These gut-induced Tregs can migrate via the bloodstream to the lungs, suppressing local inflammatory responses—a key component of immune regulation along the gut-lung axis (**Figure 1e**). Additionally, SCFAs can upregulate neutrophil chemokine receptors (e.g., CXCR1 and CXCR2) through the HDAC pathway, enhancing neutrophils’ sensitivity to chemotactic signals and promoting their migration to inflammatory sites. Concurrently, high doses of propionate and butyrate induce caspase-dependent apoptosis in normal neutrophils (67), facilitating timely clearance of functionally exhausted cells and preventing excessive inflammation.

Specifically, SCFAs synergistically maintain immune homeostasis via two core pathways: first, acting as ligands for receptors like GPR109A to drive the differentiation of naive T cells into Tregs within antigen-presenting cells, while suppressing colonic inflammation by inducing anti-inflammatory factor networks (68); second, directly enhancing acetylation levels at key gene sites such as Foxp3 by inhibiting HDAC activity, thereby synergistically amplifying Treg expansion and jointly regulating inflammatory responses (56).

Beyond regulating T cells, macrophages, and the epithelial barrier, SCFAs modulation of B lymphocytes—particularly regulatory B cells (Bregs)—has increasingly emerged as another critical link in maintaining gut-lung axis immune homeostasis. Research by Claudia Mauri’steam provides a clear mechanistic example. The study revealed significantly reduced butyrate.

levels in the intestines of rheumatoid arthritis (RA) patients and model mice, positively correlated with the frequency of circulating IL-10+ Bregs (69). Mechanistically, butyrate supplementation does not directly act on B cells; instead, it reshapes the gut microbiota, enriching bacteria such as Allobaculum and Bifidobacterium that influence tryptophan metabolism. This markedly increased levels of the serotonin metabolite 5-hydroxyindole-3-acetic acid (5-HIAA). As an endogenous ligand, 5-HIAA activates the aryl hydrocarbon receptor (AhR) on B cells surfaces. AhR activation subsequently initiates a transcriptional program that supports Bregs function, enhancing IL-10 secretion while inhibiting differentiation into plasma cells (70), thereby exerting therapeutic effects in arthritis models (69, 71) (**Figure 1f**). This study first elucidates how a microbial metabolite precisely regulates Breg function via AhR by modulating another metabolite (5-HIAA).

Beyond these indirect pathways mediated by specific metabolites, whether SCFAs can directly act on B cells through their inherent HDAC inhibitory activity—like their regulation of T cells—remains a compelling scientific question. SCFAs (particularly butyrate) are known potent histone deacetylase inhibitors (HDACi) (72). Based on this, it is hypothesized that SCFAs may directly promote Bregs differentiation by altering chromatin accessibility of genes associated with B cells differentiation and function. However, the specific role of this direct mechanism within the gut-lung axis remains to be confirmed by research.

In the context of the gut-lung axis, gut-derived SCFAs may help maintain a regulatory Bregs population in the lungs. These pulmonary Bregs subsequently suppress pathogenic T cells responses (e.g., Th2, Th17) and neutrophilic inflammation through mechanisms such as IL-10 secretion, thereby exerting protective effects in the immunopathological regulation of diseases like asthma and COPD. Thus, elucidating key pathways by which SCFAs regulate pulmonary immune homeostasis via Bregs deepens our understanding of gut-lung axis communication mechanisms.

SCFAs serve as pivotal messengers within the gut-lung axis. However, current research exhibits notable limitations. First, studies have disproportionately focused on butyrate while neglecting SCFAs ratios. In the human gut, acetate constitutes 60–70%, propionate 20–30%, and butyrate 5–10%—a ratio fundamental to maintaining microbial community homeostasis and systemic immune equilibrium. Yet, when this ratio becomes imbalanced—such as with excessively high acetate levels—whether this disrupts the pulmonary immune microenvironment and exacerbates lung inflammation remains unsupported by systematic animal or clinical research data. Secondly, it is worth noting that most current research on the SCFAs-HDAC-Treg axis originates from mouse models. While the underlying mechanisms are considered conserved in humans, species differences may exist in SCFAs types, concentrations, and tissue-specific effects. These variations must be carefully considered when translating findings from mouse models to human clinical applications. Finally, the actual concentration of SCFAs reaching the lungs via the bloodstream and whether they can attain effective anti-inflammatory thresholds requires further validation through animal experiments and clinical data. Future research should focus on the synergistic effects of SCFAs groups and their precise functions at physiological concentrations.

### 3.2 Tryptophan and its derivatives

Research has revealed that serum levels of tryptophan metabolites are significantly reduced in patients with allergic asthma (73). Intraperitoneal injection of tryptophan metabolites alleviates asthma symptoms in OVA-induced asthmatic mice while decreasing OVA-IgE and inflammatory factor levels (74). These effects may be attributed to the regulation of Th17 and Tregs (75) differentiation. Tryptophan, an essential amino acid indispensable in the human diet, and its metabolites derived from dietary sources and the gut microbiota act as ligands for aromatic receptors. These metabolites regulate various pathophysiological processes, such as immune responses, and mediate gut mucosal barrier protection through multiple pathways involving gut microbial interactions. Intestinal tryptophan metabolism involves the kynurenine (Kyn), serotonin (5-HT), and indole pathways (76). Under the action of gut symbionts, tryptophan can be converted into indole, indole-3-aldehyde (IAld), indole-3-propionic acid (IPA), indole-lactic acid (ILA), indole-3-ethanol, tryptamine (TAM), indoleacetaldehyde (IAM), indoleacrylic acid (IA), and other indole derivatives (77). The host and gut microbiota engage in competitive metabolism of tryptophan: the host produces pro-inflammatory metabolites via the Kyn pathway, while the gut microbiota generates anti-inflammatory and barrier-protective metabolites via the indole pathway. The equilibrium between these pathways directly influences immune homeostasis; disruption is closely associated with inflammatory diseases and autoimmune disorders. The mechanisms underlying this balance within the gut-lung axis remain poorly understood, and future research will focus on comparing the differential effects of various indole derivatives.

**Figure 2:**
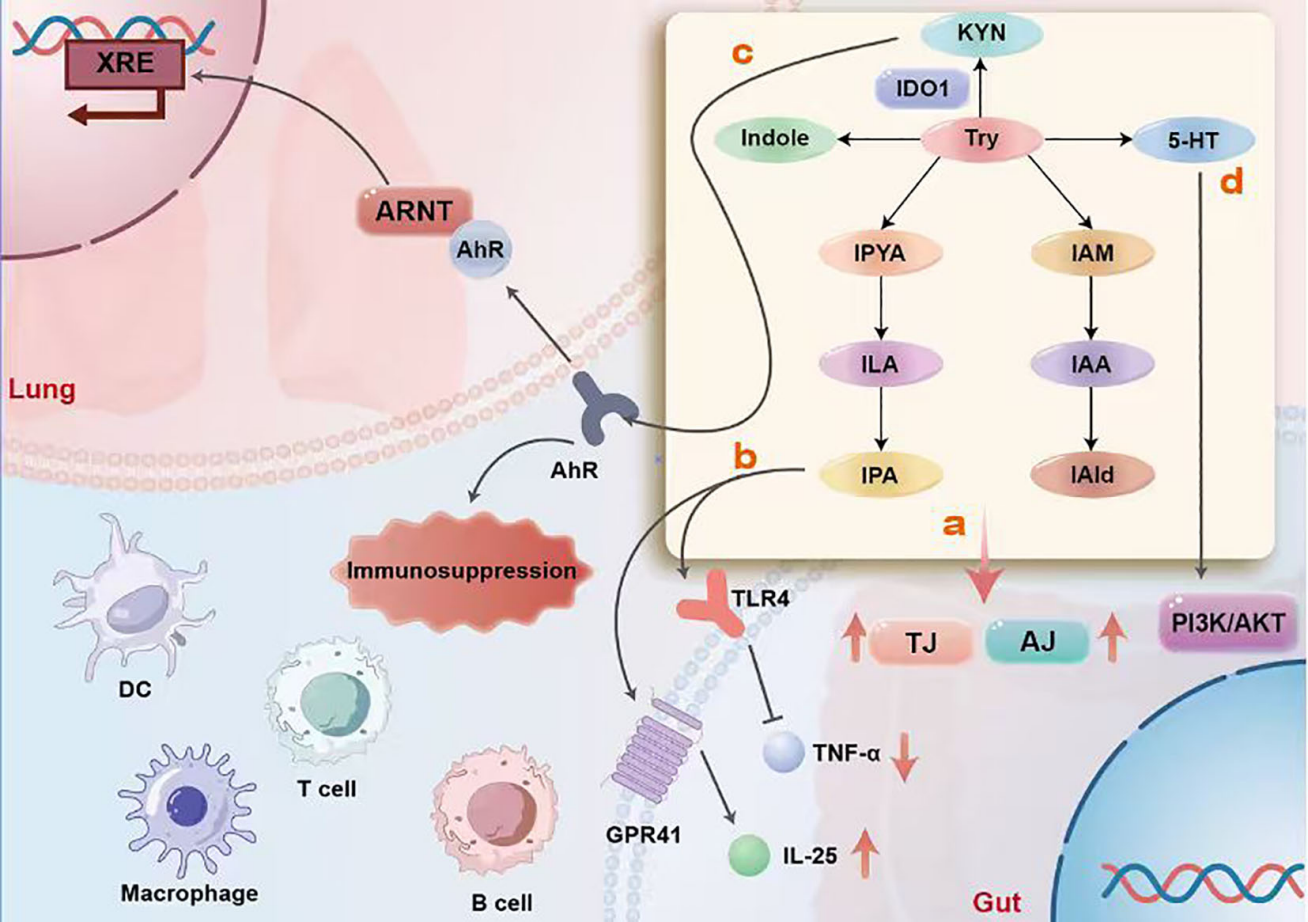
The schematic illustrates how tryptophan metabolites originating from the gut reach the lungs via the bloodstream and regulate immune homeostasis through four core pathways, divided into two major modules: the gut (right side) and the lungs (left side). In the gut, microorganisms convert tryptophan into indole and its derivatives (such as IAId and IAA). Upon entering the circulatory system and reaching the lungs, these substances activate AhR as endogenous ligands. Activated AhR forms a complex with ARNT proteins in the cell nucleus, binds to XRE response elements, and initiates a key immunosuppressive transcriptional program. This program modulates the functions of DCs, macrophages, T cells, and B cells, ultimately inducing an immunosuppressive state to alleviate excessive lung inflammation **(c)**. 5-HT produced by gut microbial metabolism enters the circulation and acts on pulmonary cells. It modulates the balance between immune cell function and inflammatory responses in the lung by activating the intracellular PI3K/AKT signaling pathway **(d)**. Multiple tryptophan metabolites exert synergistic effects through distinct receptor mechanisms. Some metabolites participate in immunoregulation by activating receptors such as GPR41; while others exert dual effects by acting on TLR4 to suppress pro-inflammatory TNF-α production and promote anti-inflammatory IL-25 release **(b)**. Furthermore, these metabolites directly strengthen TJ and AJ between intestinal epithelial cells, reinforcing the physical barrier to prevent translocation of harmful substances and maintain systemic immune stability at its source **(a)**.

Early studies indicate that compared to non-specific pathogen-free mice, germ-free mice exhibit reduced mRNA expression of tight junction (TJ) or adherens junction (AJ) molecules in colonic epithelium, alongside significantly lower indole levels. Treatment with indole capsules reverses this phenomenon (78), confirming that tryptophan and its derivatives promote the establishment of the intestinal epithelial barrier *in vivo* (**Figure 2a**). Similarly, IPA, a metabolite of tryptophan and an endogenous ligand for the pregnane X receptor (PXR), modulates luminal sensing and signaling pathways via toll-like receptor 4 (TLR4) to regulate these pathways (79). It downregulates TNF-α expression in intestinal cells within mouse models of intestinal inflammation, while upregulating the expression levels of tight junction proteins. Furthermore, it activates tuft cells via the GRP41 pathway, increasing IL-25 secretion and thereby safeguarding intestinal barrier integrity (**Figure 2b**).

Upon binding to Toll-like receptors (TLRs) on the surfaces of immune cells, gut microbiota and their metabolites induce the upregulation of indoleamine 2,3-dioxygenase 1 (IDO1) expression in host cells (80, 81), thereby stimulating IDO1 activity (82). This promotes the conversion of tryptophan into Kyn. Kyn can regulate immune cell differentiation and function by activating the AhR in pulmonary endothelial cells. This includes promoting Treg generation and suppressing excessive activation of effector T cells, thereby maintaining immune homeostasis in the gut and lungs. In the gut, the Kyn-AhR signaling pathway helps prevent excessive immune responses by gut immune cells against commensal bacteria; In the lungs, this pathway modulates immune responses to pathogens or allergens, reducing inflammatory reactions. Indole and its derivatives also activate AhR. For instance, indolepyruvate stimulates AhR to decrease Th1 cells and increase Tr1 cells in colonic lymphoid tissue, thereby resolving chronic inflammation in a T cell-mediated colitis mouse model. Indole-3-pyruvate also reduces the ability of mesenteric lymph node (MLN) DCs to induce Th1 cell differentiation via AhR, increasing anti-inflammatory CD103_(+)_ CD11b_(-)_ DCs in MLNs (83). Indole-3-carboxaldehyde absorbed into the circulation from the gut specifically binds to AhR on pulmonary vascular endothelial cells. Upon AhR activation, it dissociates from heat shock proteins and forms heterodimers with the Aromatic Receptor Nuclear Translocation Protein (ARNT). This heterodimer enters the cell nucleus and binds to specific DNA sequences, namely the exogenous response element (XRE), thereby regulating the transcription of downstream target genes. This affects signaling molecules downstream of the Apelin-APJ signaling pathway, such as by modulating intracellular levels of second messengers (e.g., cAMP, Ca²^+^) or influencing protein kinase activity, thereby activating related signaling pathways (**Figure 2c**).

Sputum from cystic fibrosis patients lacks 5-HT. Supplementing 5-HT restores the disrupted tryptophan metabolism observed in the disease. Tryptophan is converted to 5-hydroxy tryptophan by tryptophan hydroxylase, which is then decarboxylated by decarboxylase to produce 5-HT. 5-HT promotes proliferation and repair of intestinal epithelial cells. It acts on 5-HT receptors in intestinal epithelial cells, activating intracellular signaling pathways such as the PI3K-AKT pathway (**Figure 2d**). This facilitates continuous renewal of intestinal epithelial cells, maintaining mucosal integrity while sustaining initiation of the IDO1/Kyn protective pathway and limiting the indole/AHR pathway to promote pathogen clearance and maintain immune homeostasis (84). It also suppresses the function of immune cells, including macrophages, T/B lymphocytes, and antigen-presenting cells.

### 3.3 PAs

**Figure 3:**
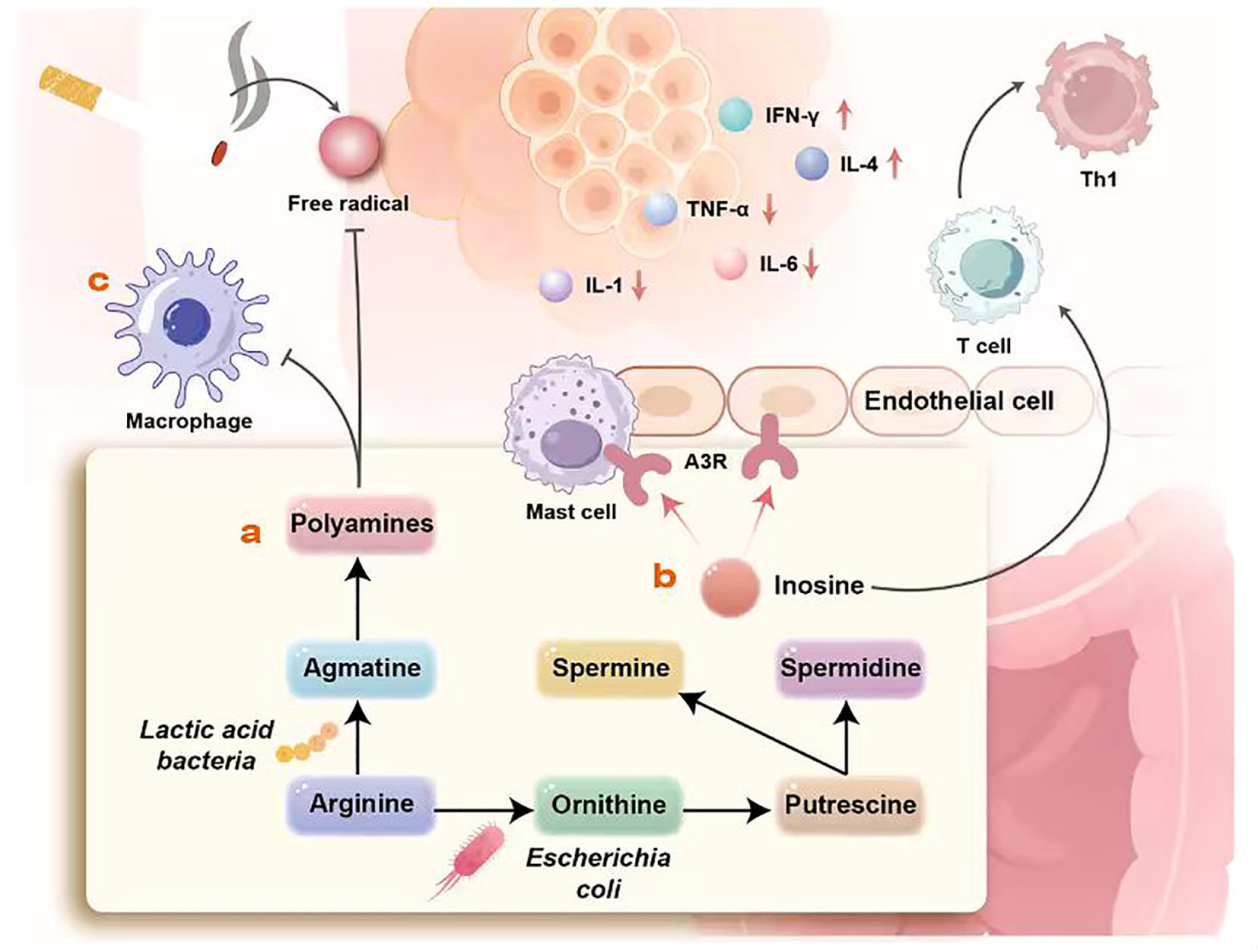
This figure systematically elucidates the molecular mechanism by which gut microbiota metabolize arginine to produce bioactive substances such as PAs, thereby influencing pulmonary immune homeostasis. It reveals how environmental factors like smoking interfere with this process by inducing free radicals. The schematic is clearly divided into two major modules: the gut (lower section) and the lungs (upper section). It illustrates how gut-derived PAs and their related metabolites reach the lungs via the bloodstream, where they regulate immune responses through three core pathways. Within the gut, microorganisms like E. coli convert arginine into ornithine, which is further metabolized into PAs (including spermine and spermidine). These PAs enter systemic circulation and reach the lungs. In the lungs, PAs induce free radical generation. This process is exacerbated by factors like smoking. Excessive free radicals damage lung cells through oxidative stress mechanisms, promote pro-inflammatory states, and alter key cytokine levels **(a)**. Inosine, a metabolite associated with polyamine metabolism, plays a crucial immunoregulatory role in the lungs. It modulates the activity of mast cells and endothelial cells by activating A3R receptors on their surfaces. Concurrently, this pathway influences the differentiation of naive T cells, promoting their polarization toward a Th1 phenotype, thereby directing the orientation of the pulmonary adaptive immune response **(b)**. In the gut, lactic acid bacteria convert arginine into Imidazolidinyl urea. Upon reaching the lungs, Imidazolidinyl urea primarily acts on alveolar macrophages to regulate their functional state. The regulated macrophages exhibit reduced secretion of proinflammatory cytokines (such as TNF-α, IL-1, IL-6), while levels of cytokines like IFN-γ and IL-4 are relatively elevated, thereby establishing an anti-inflammatory and immunoregulatory environment in the lungs **(c)**.

Multiple microorganisms in the gut possess the ability to synthesize PAs. For example, bacteria such as E. coli can convert arginine into ornithine through specific metabolic pathways. Ornithine is then converted into putrescine by ornithine decarboxylase, which can further transform into spermine and spermidine (85) (**Figure 3a**). Additionally, certain lactic acid bacteria can utilize arginine to produce guanidine, which undergoes a series of reactions to generate PAs. These microbially derived PAs exert significant regulatory effects on pulmonary immunity, particularly on the function of alveolar macrophages. PAs not only enhance the phagocytic activity of alveolar macrophages against pathogens but also regulate their release of cytokines and inflammatory mediators, such as TNF-α and IL-1. In smoking-induced pulmonary inflammation, PAs also exhibit antioxidant effects, mitigating oxidative damage to alveolar macrophages induced by cigarette smoke by scavenging free radicals, thereby reducing the severity of inflammatory responses (**Figure 3b**). Another study revealed the pivotal role of inosine derived from Bifidobacterium pseudofasciolopium in host defense against non-tuberculous mycobacterial (NTM) infection (86). Its protective mechanism is dual-pronged. Upon entering the systemic circulation, inosine stimulates effector T cells to produce more IFN-γ and promotes Th1 cell differentiation, thereby enhancing cellular immunity and effectively suppressing NTM infection. Simultaneously, inosine binds to A3 adenosine receptors on pulmonary mast cells and alveolar epithelial cells (AECs). It reduces tissue damage by downregulating pro-inflammatory factors, such as TNF-α, IL-1, and IL-6, while elevating the anti-inflammatory factor IL-4 (87), thereby exerting a protective effect (**Figure 3c**).

In summary, based on previous research, gut microbiota metabolites, including SCFAs, tryptophan metabolites, and PAs, can regulate Tregs balance and participate in cytokine crosstalk, thereby promoting healthy immune responses. Along the gut-lung axis, immune cell signaling between the two organs, as well as the direct effects of gut metabolites, can modulate the body’s immune environment. Disruptions in the gut microbiota may consequently influence pulmonary inflammation. Therefore, delving into the mechanisms underlying gut microbial dysbiosis opens new avenues for developing targeted therapeutic strategies aimed at the gut microbiome to intervene in and treat immune-mediated lung diseases.

## 4 The impact of gut microbiota dysbiosis on lung diseases

The microbiota maintains a dynamic equilibrium within a healthy organism. Once this balance is disrupted, it may lead to disease by altering the host’s immune function. Microbiota dysbiosis typically refers to abnormal changes in the composition or function of the microbial community. At the genus level, dysbiosis may manifest as a reduction in beneficial bacteria, such as Bifidobacterium, Lactobacillus, and Prevotella, or an increase in potentially pathogenic bacteria, like Streptococcus and Enterococcus, often accompanied by decreased community diversity or abnormal proportions of phyla. Microbiome diversity is typically assessed through two dimensions: α-diversity and β-diversity. α-diversity measures species richness and evenness within individual samples; reductions in common metrics (e.g., Shannon index, Chao 1 index) are often associated with disease states. β-diversity, however, compares overall differences in microbial community composition between samples. Visualized through multivariate statistical.

methods, such as principal coordinate analysis (PCoA), it effectively reveals significant separation in microbial structure between diseased groups and healthy controls. It is noteworthy that the lung and gut microbiomes exhibit close bidirectional crosstalk via the gut-lung axis (34), and imbalances occurring in one organ can affect the other (33, 88). For instance, early life represents a critical period for gut microbiota colonization. During this phase, reduced specificity of four bacterial genera—Faecalibacterium, Lachnospira, Veillonella, and Rothia—increases the subsequent risk of developing lung diseases such as asthma (12).

The gut microbiota is essential for immune responses (89). A healthy gut microbiota serves as the body’s innate immune barrier, effectively preventing harmful microorganisms from penetrating the intestinal wall into the bloodstream. Research by Schuijt et al. revealed that germ-free mice not only exhibited accelerated bacterial spread, severe inflammation, and organ failure, but also demonstrated impaired phagocytic capacity in alveolar macrophages, along with compromised innate and adaptive antiviral immune responses (90, 91). Furthermore, disruption of gut microbial balance by antibiotics and other factors impairs the ability of pulmonary macrophages to produce type I and type II IFNs. This significantly reduces mice’s capacity to limit viral replication and clear the influenza virus after infection (92). These findings collectively demonstrate that a stable gut microbiota is essential for maintaining effective pulmonary immune defense.

Distinct pulmonary diseases are frequently associated with characteristic dysbiosis of the gut microbiota (37, 57). Studies reveal a reduced abundance of the Firmicutes phylum and an increased abundance of the Proteobacteria phylum in the guts of patients with COPD. Concurrently, beneficial bacteria, such as the Bifidobacterium and Lactobacillus genera, decrease in number (93–95). This imbalance may exacerbate systemic inflammation by reducing the production of SCFAs. Compared to healthy individuals, COVID-19 patients exhibit significantly reduced gut bacterial diversity, characterized by a substantially increased relative abundance of opportunistic pathogens, such as Streptococcus, Lactobacillus, Micrococcus, and Actinomyces, alongside decreased proportions of beneficial symbionts (96). Such disruption may impair immune responses and contribute to disease progression.

### 4.1 Asthma

Numerous cohort studies have confirmed that early-life gut microbiota dysbiosis is a significant risk factor for the development of subsequent asthma (97–99). Moreover, asthma patients typically exhibit lower gut microbiota diversity than healthy individuals, with shared characteristics across different regions and populations. For instance, in Canadian children at high risk for asthma, the relative abundance of Spirochaeta, Microspira, Faecalibacterium, and Ruminococcus was significantly reduced, accompanied by decreased acetate levels and abnormal regulation of gut-liver metabolites (12). Similarly, increased relative abundance of Streptococcus and Bacteroides species alongside decreased Bifidobacterium and Nodularibacter species in fecal samples from Ecuadorian children at 3 months of age was associated with a higher risk of atopy and wheezing by age 5 (100). Among US newborns, individuals with low relative abundances of Bifidobacterium, Akkermansia, and Faecalibacterium genera, coupled with high relative abundances of Candida and Rhodotorula, exhibited the highest risk of developing atopy and asthma (101). These findings collectively demonstrate that the absence of specific protective bacterial genera and reduced gut diversity in early life are significantly associated with an increased risk of asthma.

From the perspective of gut microbial metabolites, SCFAs produced by gut microbes fermenting dietary fiber exert a clear protective effect against asthma (7, 52). Children with high fecal butyrate and propionate levels at age 1 exhibited lower subsequent risks of atopic sensitization and asthma (102). To elucidate the influence of the gut microbiota on the distal lung, numerous studies have utilized mouse models. These investigations suggest that microbial metabolites, such as SCFAs, may promote Treg expansion and IL-10 production by inhibiting epigenetic pathways, including histone deacetylation, thereby alleviating airway inflammation (103). However, not all microbial metabolites exert protective effects. For example, increased levels of histamine-producing bacteria in the feces of asthma patients correlate with disease severity. In various experimental models, bacterial-derived histamine and lipid metabolites, such as 12,13-diHOME, exhibit complex effects on asthma inflammatory responses and may even promote inflammation (104).

Furthermore, clinical evidence suggests that gut dysbiosis primarily influences asthma through two mechanisms: abnormal immune regulation and impaired intestinal barrier function. Dysbiosis disrupts immune regulation by enhancing Th2 cell responses, increasing the secretion of pro-inflammatory cytokines, triggering airway inflammation and hyperresponsiveness, and exacerbating asthma symptoms. Simultaneously, it compromises the intestinal barrier, allowing harmful substances and allergens to enter the bloodstream or tissues, thereby activating the immune system and further driving the development of asthma.

More critically, the gut-lung axis operates in a bidirectional manner. Persistent pulmonary inflammation caused by asthma can reciprocally disrupt the gut environment by inducing systemic inflammation, altering neuroendocrine function, and affecting the migration of immune cells. This leads to impaired intestinal barrier function and a further reduction in microbial diversity. This secondary gut microbiota dysregulation may then exacerbate systemic inflammation, creating a vicious cycle that promotes asthma chronicity and severity. Collectively, human epidemiological studies and animal models reveal the pivotal role of the gut-lung axis in asthma pathogenesis.

### 4.2 Acute respiratory distress syndrome

Acute respiratory distress syndrome (ARDS) is a severe respiratory failure disorder characterized by high incidence and mortality rates (105). Recent studies have revealed widespread dysbiosis of the gut microbiota in ARDS patients, primarily attributed to systemic dysregulation of inflammation and disruption of the intestinal barrier. During ARDS onset, levels of proinflammatory factors such as IL-1β and TNF-α surge dramatically in patients’ bronchoalveolar lavage fluid (BALF) and plasma. Once these factors enter the bloodstream, they activate pulmonary immune cells, triggering systemic inflammatory dysregulation (106). Concurrently, gut dysbiosis severely compromises the intestinal barrier, causing abnormalities in tight junction proteins. This includes the upregulation of junctional adhesion molecules (JAM) and claudin 2, alongside the downregulation of claudin 5 (107). Additionally, the distribution of claudin 1, 3, 4, 5, and 8 becomes disorganized, significantly increasing intestinal permeability (108, 109). This allows harmful substances, such as bacteria and endotoxins, to breach the barrier and enter the systemic circulation, triggering a systemic inflammatory response that ultimately increases mortality.

Mechanical ventilation (MV) remains a core therapeutic approach for ARDS (110); however, it simultaneously induces ventilator-induced lung injury and exacerbates both local and systemic inflammation. Notably, this ventilation-induced inflammatory response reciprocally impacts intestinal blood flow and barrier function, altering the gut environment. This further aggravates dysbiosis and barrier damage, complicating the clinical course. Consequently, interventions targeting the gut microbiota—such as probiotic administration or fecal microbiota transplantation (FMT)—may improve ARDS outcomes by repairing the intestinal barrier, suppressing bacterial translocation, and modulating the immune response.

### 4.3 COPD

COPD is a lung disorder characterized by airflow limitation. Currently, COPD ranks first among chronic respiratory diseases in all-cause mortality, causing over 3 million deaths globally each year (111, 112). Therefore, in-depth research into the pathogenesis of COPD is urgently needed.

Smoking is a major risk factor for COPD. Harmful substances in tobacco smoke, such as tar, nicotine, and carbon monoxide, directly damage airway epithelial cells, disrupt the mucociliary.

clearance system, increase epithelial reactive oxygen species (ROS) levels, heighten permeability, and activate innate immune cells in the lungs to secrete large amounts of pro-inflammatory factors (113), thereby triggering chronic inflammation and airflow limitation. Smoking not only exacerbates COPD symptoms and accelerates lung function decline (114) but may also alter the gut microbiota, promoting biofilm formation by specific bacterial genera, such as Streptococcus, and enhancing their intestinal colonization capacity (115).

Growing evidence indicates that gut microbiota dysbiosis is also a significant factor influencing COPD pathophysiology (116, 117). On the one hand, gut microbiota interacts with DCs, inducing innate lymphoid cells in the gut to express the chemotactic receptor CCR4, which enables their migration to the lungs and increases susceptibility to respiratory infections (118). On the other hand, disruption of the gut microbiome leads to elevated levels of inflammatory mediators, such as IL-17A, IL-17F, and TNF-α, which then reach the lungs via the bloodstream, recruiting and activating neutrophils and macrophages to exacerbate pulmonary inflammation. Concurrently, gut and lung microbiota dysbiosis can activate NLRP3 inflammasomes, which mediate the secretion of proinflammatory cytokines such as IL-1β and IL-18, thereby further aggravating COPD.

Analysis via 16S rRNA gene sequencing reveals significant differences in gut microbiota between patients with COPD and healthy individuals, with these disparities widening as disease severity increases. Patients commonly exhibit reduced gut microbial diversity, a decreased relative abundance of the Bacteroidetes phylum, an increased relative abundance of the Firmicutes phylum, imbalanced ratios between the two phyla, and significantly lower levels of SCFAs, such as acetate, isobutyrate, and isovalerate. Total SCFAs levels are also lower than in healthy individuals (119). Collectively, these alterations compromise the integrity of the intestinal epithelial barrier, allowing harmful substances, such as endotoxins, to enter the bloodstream. This triggers systemic inflammation and oxidative stress. These substances reach the lungs via the bloodstream, continuously activating immune cells and exacerbating the pathological progression of COPD. Concurrently, the decline in SCFAs levels weakens their role in inducing Tregs, further diminishing the gut’s innate immune protection for the lungs.

Population-based epidemiological studies confirm that COPD frequently coexists with chronic gastrointestinal diseases, demonstrating bidirectionality in the gut-lung axis. The prevalence of IBD among COPD patients is significantly higher than in the general population (120, 121). Conversely, individuals with IBD may face a higher risk of developing COPD compared to healthy individuals. COPD patients, due to impaired lung function and long-term use of medications such as corticosteroids and aminophylline, may experience intestinal hypoxia, mucosal damage, and dysbiosis (122). This, in turn, increases intestinal epithelial permeability, mediates inflammatory responses, and exacerbates symptoms of pulmonary disease, further demonstrating the impact of the gut-lung axis on COPD.

### 4.4 Pulmonary fibrosis

PF is a disease characterized by abnormal proliferation of fibrous tissue within the pulmonary interstitium and destruction of alveolar structures (123), with IPF being the most common form. Its core pathogenesis involves the abnormal repair of alveolar epithelial cells following repeated injury (124), which releases pro-fibrotic signals, such as TGF-β (125), to activate pulmonary fibroblasts and induce their transformation into myofibroblasts. These cells resist apoptosis, leading to excessive extracellular matrix deposition (126) and destructive remodeling. Concurrently, the dysregulation of immune cells, such as M2 macrophages and ILC2 cells, exacerbates this process (127), ultimately leading to irreversible pulmonary scarring.

PF not only causes irreversible lung function impairment but also disrupts gut microbiota composition and associated metabolites through bidirectional gut-lung axis communication (123). Studies on early silicosis patients reveal that their primary pathological feature—progressive PF—is accompanied by significantly reduced operational taxonomic unit (OTU) numbers and Shannon diversity indices in gut microbiota compared to healthy individuals. Abnormal changes in gut bacteria, such as Proteobacteria, Verrucomicrobia, Firmicutes, and Actinobacteria, are primarily involved in biological processes such as cell membrane synthesis, amino acid transport and metabolism, post-translational modification, inorganic ion transport, and nucleotide transport and metabolism (128). At the genus level, gut microbiota dysbiosis in PF patients is characterized by reduced bacterial richness and diversity. Compared to healthy individuals, patients with chronic pulmonary fibrosis exhibit a markedly increased abundance of Staphylococcus, Streptococcus, and Veillonella species, while Bacteroides, Bifidobacterium adolescentis, and Propionibacterium species are significantly depleted (129). This characteristic dysbiosis drives the progression of PF through two primary pathways. Gut dysbiosis promotes the differentiation of pro-inflammatory Th17 cells while reducing the number of Tregs. These immune cells migrate through the bloodstream to the lungs, releasing pro-inflammatory factors such as IL-17 (130) to create a persistent inflammatory environment that activates fibroblasts and promotes fibrosis. Additionally, gut dysbiosis alters the spectrum of microbial metabolites, primarily characterized by reduced production of anti-inflammatory SCFAs (e.g., butyrate) and increased levels of potentially pro-inflammatory metabolites (e.g., TMAO). These metabolites act on the lungs via the bloodstream, further exacerbating immune imbalance and directly or indirectly promoting the development of fibrosis. Thus, the gut microbiota not only offers new insights into understanding the pathogenesis of PF but also provides potential targets for developing novel microbiome-based biomarkers and intervention strategies.

## 5 Therapeutic interventions

Restoring microbial balance through targeted interventions offers a highly promising new therapeutic approach for lung disorders driven by gut dysbiosis (131). Research into interventions such as probiotics, FMT, and CRISPR-Cas systems is crucial. Particular emphasis should be placed on analyzing immune and clinical responses post-intervention, applying personalized medicine, and creating effective therapies tailored to the pathogenic mechanisms present in specific patients or patient groups.

### 5.1 Probiotics

Probiotics refer to live microorganisms that exert beneficial effects on the host, primarily including Lactobacillus, Bifidobacterium, and certain yeasts. They promote gut microbiota balance through mechanisms such as nutrient competition, production of antimicrobial substances, and immune modulation (132, 133). In recent years, based on the gut-lung axis theory, probiotic intervention has emerged as a highly regarded new strategy in the prevention and treatment of pulmonary diseases.

In terms of immune modulation, probiotics enhance intestinal barrier function, mitigate excessive inflammatory responses, boost natural killer (NK) cell activity, promote Th1-type immune responses (134), and stimulate IgA-dependent mucosal immune responses in the small intestine and lungs. This reduces airway eosinophil infiltration and hyperresponsiveness, thereby decreasing the risk of respiratory infections and allergies.

Probiotics can modify the gut microbiota, emerging as a novel therapeutic target for the prevention and treatment of lung disease (135). Extensive research has demonstrated their potential. For instance, in mouse models, the depletion or absence of segmented filamentous bacteria (SFB) leads to impaired immune responses and worsened outcomes following respiratory infections (90, 136, 137), whereas supplementation with SFB enhances resistance to Staphylococcus aureus pneumonia and viral lung infections (138, 139). In a study involving asthma patients, all subjects received beclomethasone, with one group additionally receiving probiotics containing Lactobacillus. The probiotic group demonstrated increased asthma control test scores, reduced symptom counts, and improved peak expiratory flow rates. Similar findings from multiple studies indicate that probiotics [such as Lactobacillus and Bifidobacterium (140–143)] demonstrate favorable therapeutic effects for pulmonary diseases like asthma and COPD (144, 145). For instance, oral administration of Bifidobacterium promotes the generation of Th1 and Tregs by regulating T cell polarization. On the one hand, Th1 cells upregulate Th1-type immunoregulatory factors, such as IFN-γ and IL-12, in the lungs to suppress Th2 cell function. On the other hand, Tregs directly inhibit Th2 and Th17 mediated inflammatory responses by secreting factors such as IL-10 (146), jointly inducing immune tolerance. This improves the characteristic immune imbalance in asthma and exerts therapeutic effects. Furthermore, oral administration of Lactobacillus GG (LGG) alleviates asthma by reducing the expression levels of alveolar lavage fluid and serum matrix metalloproteinase-9 (MMP-9), thereby inhibiting inflammatory cell infiltration in the lungs (147). In COPD models, the probiotic Pediococcus pentosaceus SMM914 modulates the gut microbiota, promotes the production of SCFAs and antioxidant metabolites, and synthesizes specific tryptophan metabolites to enhance antioxidant and anti-inflammatory activity. This reduces M1 macrophage polarization, alleviating pulmonary oxidative stress and inflammation (148). Oral probiotic mixtures can reverse gut microbiota dysbiosis induced by respiratory syncytial virus (RSV) infection and restore pulmonary microbial composition, exerting protective effects through the microbiota-alveolar macrophage axis (149).

However, clinical application of probiotics in pulmonary diseases remains challenging. Efficacy is inconsistent, with some studies showing no significant difference between probiotic and placebo groups in improving core pulmonary function metrics (e.g., FVC%, FEV1%), reducing disease exacerbations, or influencing gut health biomarkers (150). These inconsistent findings underscore that probiotics should not be viewed as standalone therapies replacing conventional medications. A more accurate characterization is that specific probiotics may function as biological response modifiers, exerting auxiliary regulatory effects on patients’ immune function, systemic inflammation levels, or infection risk via the gut-lung axis, rather than directly reversing pathology. Currently, most evidence stems from animal studies and limited-scale human trials. To achieve precise clinical application, large-scale, multicenter, randomized, double-blind controlled trials are still required to clarify their efficacy, safety, and optimal application strategies.

### 5.2 FMT

FMT involves transferring the gut microbiota from a healthy donor to a recipient. It represents the most direct method for reconstructing and restoring the gut microbiota, encompassing nearly all its members and functions (151). This procedure is commonly employed to treat certain intestinal and extraintestinal diseases (152). In animal studies, gastric lavage is commonly used to ensure the direct delivery of donor fecal microbiota into the recipient’s gastrointestinal tract, thereby facilitating microbial colonization and functional regulation (119, 153). This method is currently most widely applied in treating clostridioides difficile infection (CDI) (154), with substantial research and promising outcomes also emerging in IBD and irritable bowel syndrome (IBS). Its application in the treatment of pulmonary disease remains exploratory but shows promise. For instance, in asthma rat models, FMT improved airway function, significantly restored lung tissue pathology, reduced alveolar wall thickening, decreased inflammatory cell infiltration, and markedly lowered collagen fiber deposition (155). In antibiotic-treated rat models of pulmonary arterial hypertension, FMT alleviated hypoxia-induced abnormalities in cardiopulmonary hemodynamics (153). Similarly, in LPS-induced ARDS mouse models, the transplantation of fecal microbiota from surviving mice significantly reduced the levels of inflammatory mediators (TNF-α, IL-1β, and IL-6) in lung tissue and bronchoalveolar lavage fluid. However, the safety of applying FMT to critical illnesses like ARDS raises significant concerns. A rescue FMT study involving 18 ICU patients revealed multiple cases of Enterobacteriaceae or Enterococcus bloodstream infections occurring before or after FMT, suggesting ICU populations are at high risk for FMT-associated bacteremia (156). This stands in stark contrast to the anti-inflammatory benefits observed in animal studies. Therefore, the safety and efficacy of FMT for ARDS remain controversial. To ensure its safety and effectiveness, detailed and comprehensive research must be conducted on disease-specific aspects, including testing of blood and feces from FMT donors, selection of fecal transplant preparations, preparation methods, storage conditions, and quality control.

### 5.3 CRISPR-Cas systems

Traditional microbiome intervention strategies (such as probiotics and FMT) face fundamental limitations in regulating complex ecosystems due to their lack of precision, making it difficult to achieve targeted manipulation of specific bacterial species or functions. The CRISPR-Cas system, a revolutionary and highly efficient gene editing tool (157), enables the specific editing of microbial genes for precise regulation (158), offering a novel approach to addressing this challenge.

Targeted gene editing of the gut microbiota using the CRISPR system provides novel strategies for deepening understanding of gut-lung axis mechanisms and advancing its clinical application in microbiome therapies for specific diseases. Its application primarily relies on two strategies: first, an *in vitro* editing approach, where specific bacteria (e.g., E. coli) are isolated and cultured, then CRISPR systems are used to knockout virulence genes or insert therapeutic genes before reintroducing them into the gut for colonization; second, an *in vivo* editing strategy, which involves developing microbial vectors or synthetic delivery systems to precisely deliver CRISPR components to target bacteria within the gut, enabling *in situ* genome editing.

Significant progress has been made in this field. For instance, phage-mediated delivery of CRISPR-Cas9 has enabled targeted elimination of Clostridium difficile toxin genes or essential genes without disrupting the commensal microbiota (159). Editing genes in species such as Bacteroides holds promise for precisely regulating the production of metabolites like SCFAs, offering potential avenues for studying the mechanisms of the gut-lung axis and treating diseases like asthma and COPD (160). For intestinal diseases, an oral multistage delivery system based on CRISPR-Cas9 has been developed. Utilizing calcium alginate microspheres for protection and engineered bacterial outer membrane vesicles for targeted delivery, this system directs RNP to immune cells at sites of intestinal inflammation, enabling efficient editing of TNF-α and offering a novel therapeutic strategy for inflammatory bowel disease (161). Under competitive colonization conditions with non-fluorescent E. coli strains, phage-delivered CRISPR–Cas9 successfully mediated gene knockout by deleting a fluorescent marker gene in E. coli strains within the gut microbiome (162).

In summary, the CRISPR system demonstrates broad potential in regulating the gut microbiome. These studies not only expand the application scope of CRISPR technology within the microbiome but also provide novel solutions for personalized medicine, antimicrobial therapy, and interventions aimed at improving gut health. Future efforts will focus on developing more efficient and safe targeted delivery systems, as well as investigating microbiome therapies tailored to specific diseases.

## 6 Discussion

This review systematically elucidates the core role of the gut microbiota in pulmonary immune homeostasis and disease via the gut-lung axis, establishing the messenger functions of key metabolites such as SCFAs, tryptophan metabolites, and PAs. However, despite deepening mechanistic exploration, translating research findings in this field into mature clinical applications still faces several major challenges and unresolved questions.

The primary issue lies in the insufficient depth and systematic nature of mechanism studies. Current research predominantly focuses on individual metabolites or isolated immune pathways, such as the SCFAs-GPR41/43-Treg axis or the tryptophan-AhR axis. Yet under physiological conditions, these pathways do not operate in isolation but form a complex, interwoven network. For instance, SCFAs can influence the expression of enzymes involved in tryptophan metabolism through HDAC inhibition pathways, while polyamine synthesis is closely linked to arginine metabolism. The cross-talk between these metabolic networks and their synergistic or antagonistic effects within the gut-lung axis remains poorly understood.

Furthermore, species differences between animal models and human physiology present a fundamental bottleneck for translating basic research into clinical applications. Common mouse asthma models fail to replicate the heterogeneity of human disease and exhibit significant disparities in gut microbiota composition, immune receptor expression, and metabolite kinetics. Most current evidence regarding SCFAs mechanisms involving HDAC inhibition originates from mouse studies, necessitating validation of their physiological relevance in humans.

Beyond correlational observations, the directionality of causality remains a central controversy in this field. While multiple studies confirm close associations between pulmonary diseases and gut dysbiosis, whether these correlations represent causation or disease-driven secondary phenomena requires further investigation (163). Taking COPD and PF as examples, whether the accompanying dysbiosis is a primary driver of disease progression or a consequence of chronic hypoxia, systemic inflammation, or drug interventions requires clarification through prospective cohort studies and mechanism-driven intervention trials.

Clinically, translational challenges include inconsistent efficacy and safety concerns. The variable outcomes of probiotics and FMT in clinical trials underscore the complexity of microbiome interventions. Efficacy may be significantly influenced by host factors such as initial microbiota composition and genetic background. Concurrently, the potential risk of bacteremia associated with FMT, along with the targeting precision and long-term safety of emerging technologies like CRISPR-Cas, represent critical barriers requiring rigorous evaluation for clinical translation.

Finally, technical bottlenecks persist. Current microbiome research heavily relies on 16S rRNA sequencing, which offers limited species-level resolution and functional prediction capabilities. While metagenomics provides richer information, efficient *in vivo* real-time monitoring technologies for the true metabolic activity and functional state of microbial communities remain lacking.

## 7 Conclusion and outlook

In summary, the gut microbiota and its metabolites serve as key messengers in the gut-lung axis, playing pivotal roles in maintaining pulmonary immune homeostasis and preventing disease. This review elucidates how key metabolites—including SCFAs, tryptophan metabolites, and PAs—profoundly influence the progression of diseases such as asthma, ARDS, COPD, and PF by regulating immune and barrier functions. However, as discussed, this field still faces significant challenges in understanding mechanistic networks, species differences, causal relationships, clinical translation, and technical approaches.

Future efforts should vigorously promote interdisciplinary collaboration to systematically integrate methodologies and technologies from microbiology, immunology, bioinformatics, and clinical medicine. This will collectively advance gut-lung axis research from mechanism elucidation toward precision treatment. Technologically, non-invasive sampling and multi-omics integration strategies should be developed, such as combining fecal, salivary, and breath sample analysis with metagenomic sequencing, portable devices, and artificial intelligence algorithms to comprehensively decipher bidirectional gut-lung regulatory networks. Simultaneously, we must deepen our understanding of the molecular and cellular mechanisms underlying interactions between microbial metabolites and host immunity. Advanced technologies like single-cell sequencing and organoid models should be leveraged to identify functionally significant key microbes and metabolites, providing targets for personalized interventions. Ultimately, through interdisciplinary collaboration and data integration, we will establish novel diagnostic and therapeutic strategies characterized by clear mechanisms, precise targeting, and individualization, offering innovative solutions for the prevention and treatment of respiratory diseases.
